# Proposed Scheme to Establish a Society for the Protection and Cure of Invalids in Belgium

**Published:** 1857-10-01

**Authors:** 


### PROPOSED SCHEME TO ESTABLISH A SOCIETY FOR THE PROTECTION AND CURE OF INVALIDS IN BELGIUM.

ONE of our most distinguished physicians, Doctor Parigot, has written to us on the subject of establishing a Society, the object of which would be to organize, on a large and original basis, the treatment of certain chronic diseases, which often resist individual care. We think it right to place M. Parigot's views before our readers, although we do not know whether they be capable of realization in our country: even admitting that this should not be the case, some suggestions of a practical nature may possibly arise from their perusal, and with this hope before us, we think they will be found worthy of attentive consideration.

The principle of association has become in our age the solution of every problem of general interest, and before which the efforts of individual action seem powerless. In spite of this recognised principle, no one has yet thought of applying it towards the cure of those diseases which afflict humanity. Surely an undertaking, the aim of which would be to procure every means of cure and protection, ought to deserve our unanimous sympathy.

A powerful and extensive Society alone could unite these curative means under one single administration; the difficulty consists in creating these means, and causing them to operate towards the well-being of a large number of invalids. To attain this end, an institution which might justly claim the title of a *Society for the Protection of Invalids*, would, in the first place, unite at fixed epochs the most celebrated medical men of our country, to which réunions they might invite those of Germany, England, France, Holland, and other countries. The Society would thereby acquire the sympathy of the entire medical world, whose general direction and scientific programme it could follow, leaving to those immediately interested the right of selecting those methods which they considered most suitable. At all events, there would not be found in the establishments of the Society a patient incapable of being benefited by the *Contre-expertise* plan of treatment adopted by the learned conclave, whose knowledge would have been derived from various schools.

It is desirable to notice that a locality amidst which a disease has been contracted, frequently offers, from its unhealthiness, serious impediments towards the recovery of a patient; and also that there is nothing which assists the medical treatment of a case more than a change of climate, country, and habits. But to obtain this assistance, it is necessary to be rich: consequently in an exceptional position. Who can travel in this age, without possessing a large fortune, so as to be ensured first-rate medical attendance?

The *Protecting Society* could alone effect, and at little cost, those therapeutical migrations from the towns to the country and the sea, where it would possess various establishments.

Should the objection be raised that such an enterprise is not of a nature to excite the attention of capitalists, we would answer that money has never been wanting in this country, even for doubtful speculations, when the aim was anything moral and noble, like that of the *Protective Society*.

We will not believe that the wealthy would be found wanting, should their concurrence be required. We will not believe that a Society, such as we desire to create, would fail in conciliating those two powerful agents of our century—mind and money; and in order to conciliate them, it would suffice, on the one hand, to realize prudently and wisely the curative suggestions of medical science, and on the other, to administer, with well-considered economy, our vast urban and rural estates, together with their industrial and agricultural productions.

Since 1830, Belgium has become a great European centre. Every year, at different periods, sections of scientific medical societies meet there from every part of the old and new worlds. Brussels, Ostend, and Spa also receive each year whole colonies of invalids, who come to seek amongst us either health or salutary distraction. By such persons, intelligent advice, and localities suited to their wants, their habits, and their requirements, could not fail in being appreciated.

Besides, the presence of learned foreigners would be of the greatest avail in forming congresses of medical consultations. These consultations could be held in the different establishments of the Society, and the practical fruit which must follow such réunions would serve as a line of conduct to the heads of the sanitary service. Each of them, and the patients especially, would not fail to understand the advantages of this combination. For the future, hopeless cases will be unknown, as every individual effort of mind as well as of experience would be combined for the removal of diseases which, hitherto, a restricted science has considered as incurable. Belgium would thus, again, become the first to project a mission of progress in the midst of the great surrounding nations, and she will have made a great step towards the solution of the problems which are connected with the sufferings of our humanity.

All our northern medical men prescribe generally for their patients a voyage to Ostend, and there is no one in our age incapable of appreciating the advantages of sea-baths in the treatment of many constitutional diseases. It is also generally known that the influence of sea air and water is most beneficial in the strumous affections of infancy and youth. Unhappily, the season for baths is often too short, on the one hand, to produce a radical effect in the cure of these affections; on the other, the long-continued and necessary expense alarms many persons, who are thus deprived of a heroic, but too expensive mode of cure.

To obviate these difficulties, it would be indispensable for the Society to construct in the neighbourhood of Ostend a special establishment, which could ensure baths at all seasons. It is unnecessary for us to enter here on technical details: we will content ourselves simply with remarking, that men of science have considered the undertaking as practicable, and are convinced that its execution would not necessarily occasion an enormous outlay. Large and elegant apartments, whose view would be only bounded by the horizon of the sea; others less extensive, but equally comfortable; halls for gymnastic exercises; saloons for assemblies; music; lectures, scientific as well as literary; also covered basins, in which salt water, sufficiently heated, could be adapted to the use of invalids and delicate persons, as also private bathing-rooms; finally, nothing need be omitted that can contribute to the cure or relief of the patients, either by medical treatment directed to their bodies, or by those pleasurable abstractions which exert their influence through the medium of the mind: a system, in short, which would render this establishment one of the most interesting institutions of Belgium. But there are seasons in the year when the sea and its vicinity assume in our country a cold and melancholy aspect—added to which, a too lengthened residence near it would end in producing a contrary effect to the one intended. Amongst the conditions essential to the cure of chronic diseases, will be found that of living, either in town or country, *under a warm atmosphere.* But where can we find in Belgium a mild and unvarying climate throughout the winter season? This question has been amply discussed, and partly decided by an article in the *Journal de Médecine de Bruxelles;* and it is stated that the portion of our country called Belgic Lorraine, but which the French designate Provence Belge, and which is situated at the foot and between the acclivities of the Ardennes, offers many localities where the temperature is higher and milder than that of any other part of Belgium. The cause of this difference is easily understood: these localities are, as it were, concealed by a curtain of mountains; they are protected against the north and east winds as entirely as is found to be the case with the *Downs* on the southern coast of the Isle of Wight, which affords to the traveller an artificial climate, very similar to that bordering on the Mediterranean. In the charming valleys hid in the depths of the Ardennes, covered gardens would complete the metamorphosis of the climate, and render the residences in these localities very advantageous in treating affections of the lungs, diseases which a well-understood treatment can either allay or cure. Every year we see numbers of affluent persons betake themselves to distant, but warm, climates; often they quit their families and their occupations with regret: to them, the Society would offer *at small cost*, and *only some hours' travelling*, all that they are driven to seek many hundreds of miles hence.

The *Protecting Society*, in order to accomplish its end, would be necessitated to purchase large and old domains in the provinces of Namur and Liège, which are arranged in some sort so as to accommodate persons accustomed to *la vie de château.* If the importance of external circumstances were sufficiently estimated as to their effects on the minds and spirits of certain invalids, it would be easily understood why the residences should be commodious and decorated with elegance—why arts and sciences should be cultivated—and why, above all, the usages of the highest society should always be observed. The *Protecting Society* would offer the same advantages to less exacting patients, for whom *villas* and even *cottages* could be adapted.

In this manner, the repose or amusements of the country, or even the occupations of a rustic life, might modify, according to the necessities of each individual, those diseases which had resisted other modes of treatment. In the case of each patient, and whatever might be his mental or physical condition, he would enjoy the benefit of home life, or that of retirement in the midst of the pure country air.

With such grounds for stability, the Society could not fail of becoming the complement of many "établissements de santé" in Germany and England: indeed, nothing would be better calculated to charm convalescents, and to render the change of country, habits, and language, mainly conducive towards their recovery.

The Society would retain the power of erecting, in a healthy locality near Brussels, a large institution for the education of children whose minds, whether from disease or other causes, were imperfectly developed. Medical men and special instructors would be placed at the head of this institution, of which Belgium and other countries are yet deficient. Brussels ought to be the centre of this Society. One of the large establishments near the city might be used as the starting-point for our operations. We are convinced that success would triumphantly demonstrate the practicability of a Society which could combine so many material and moral advantages.

The formation of this Society would be the realization of a new idea—that of curing *en grand*, by means which it would be impracticable to employ individually. At all events, the projector can conscientiously apply to himself those two immortal lines of Camoens, in which he changes only one single word:—

"Vereis amor da sciencia, nâo movido
De premio vil; mas alto, e quasi eterno!"

(From the *Journal de l'Independance Belge.*)

---

